# A Poly(ethylene oxide)/Lithium bis(trifluoromethanesulfonyl)imide-Coated Polypropylene Membrane for a High-Loading Lithium–Sulfur Battery

**DOI:** 10.3390/polym13040535

**Published:** 2021-02-11

**Authors:** Li-Ling Chiu, Sheng-Heng Chung

**Affiliations:** 1 Department of Materials Science and Engineering, National Cheng Kung University, Tainan 701, Taiwan; N56094570@gs.ncku.edu.tw; 2 Hierarchical Green-Energy Materials Research Center, National Cheng Kung University, Tainan 701, Taiwan

**Keywords:** lithium–sulfur battery, polysulfide, separator, gel polymer electrolyte, high active-material loading

## Abstract

In lithium–sulfur cells, the dissolution and relocation of the liquid-state active material (polysulfides) lead to fast capacity fading and low Coulombic efficiency, resulting in poor long-term electrochemical stability. To solve this problem, we synthesize a composite using a gel polymer electrolyte and a separator as a functional membrane, coated with a layer of poly(ethylene oxide) (PEO) and lithium bis(trifluoromethanesulfonyl)imide (LiTFSI). The PEO/LiTFSI-coated polypropylene membrane slows the diffusion of polysulfides and stabilizes the liquid-state active material within the cathode region of the cell, while allowing smooth lithium-ion transfer. The lithium-sulfur cells with the developed membrane demonstrate a high charge-storage capacity of 1212 mA∙h g^−1^, 981 mA∙h g^−1^, and 637 mA∙h g^−1^ at high sulfur loadings of 2 mg cm^−2^, 4 mg cm^−2^, and 6 mg cm^−2^, respectively, and maintains a high reversible capacity of 534 mA∙h g^−1^ after 200 cycles, proving its ability to block the irreversible diffusion of polysulfides and to maintain the stabilized polysulfides as the catholyte for improved electrochemical utilization and stability. As a comparison, reference and control cells fabricated using a PEO-coated polypropylene membrane and a regular separator, respectively, show a poor capacity of 662 mA∙h g^−1^ and a short cycle life of 50 cycles.

## 1. Introduction

The increasing demand for high-energy-density storage, together with the limited availability and increasing costs of the active materials used in commercial lithium-ion battery cathodes, has stimulated intense research on next-generation rechargeable battery systems that outperform lithium-ion batteries [1]. One such technology, the electrochemical lithium–sulfur battery, is receiving increasing attention: its high-capacity sulfur cathode has a high theoretical charge-storage capacity (1675 mA∙h g^−1^) and hence a high energy density (2600 W∙h kg^−1^) that is three to five times higher than that of commercial lithium-ion batteries [2]. Although it is the leading choice for future energy storage, current lithium–sulfur technology cannot satisfy long-term energy-storage requirements due to the characteristics of the materials involved [3]. Specifically, in fully charged and fully discharged states, solid-state sulfur and lithium sulfides exhibit high resistivity and thus low electronic conductivity. This results in poor electrochemical utilization and a low amount of sulfur in the cathode, limiting the development of lithium–sulfur batteries with a high energy density [4]. Moreover, during cycling, the converted solid-state active materials affect the reaction kinetics and cause irreversible capacity loss [5]. In intermediate charge and discharge states, liquid-state polysulfides that are generated in the cathode readily dissolve in the liquid electrolyte and thus diffuse out of the cathode. The dissolved polysulfides have high mobility and strong reaction activity and thus irreversibly relocate in the cell and damage the stability of the electrolyte and the electrodes [6]. These polysulfide-related problems lead to a short cycle-life and poor Coulombic efficiency, which hinder the development of high-loading sulfur cathodes, as such cathodes would need to stabilize a large amount of polysulfides during each charge and discharge reaction [7].

An effective approach to solving the foregoing issues is to modify the cell components by using a porous cathode substrate to host the active material [8,9] and a functional separator that has a multifunctional coating layer tightly bound to a commercial polymeric membrane to limit the irreversible migration of polysulfides from the cathode [10,11,12,13,14,15]. To this end, many recent studies have focused on designing multifunctional separators and their materials as strategies to enhance the electrochemical performance of lithium–sulfur batteries [14,15,16]. The coating layer is also bound to the surface of the cathode and is configured such that it blocks the fast diffusion of polysulfides and thus limits the loss of active material [17]. Moreover, as a polymeric separator is an essential component in lithium–sulfur batteries and other rechargeable batteries, synthesizing and fabricating the membrane does not add to the overall fabrication complexity [18]. Thus, the functionalization of a battery separator with a layer of coating material is a facile and inexpensive way to improve the performance of lithium–sulfur batteries. The coating improves the electrochemical characteristics of a sulfur cathode by serving as a conductive carbon coating for fast electron transfer [14,15,19,20,21,22], as a polysulfide-trapping coating for strong polysulfide stabilization [14,15,21,22,23,24], or as a catalyst coating for efficient conversion between solid- and liquid-state active materials [14,15,21,25,26,27]. Furthermore, a composite coating can realize all of the aforementioned advantages, enabling the functional separator to trap the dissolved polysulfides and subsequently endow the trapped active material with fast reaction kinetics, high polysulfide retention, and strong conversion capability [14,15,28,29,30,31,32,33]. In summary, state-of-the-art functional separators greatly improve the electrochemical utilization and stability of lithium–sulfur battery cathodes [10,11,12,13,14,15,19,20,21,22,23,24,25,26,27,28,29,30,31,32,33].

In this study, to improve the electrochemical utilization and reversibility of lithium–sulfur cells, we develop a composite functional separator composed of a gel polymer electrolyte and a polypropylene membrane. This functional separator is fabricated using a layer of a mixture containing poly(ethylene oxide) (PEO) and lithium bis(trifluoromethanesulfonyl)imide (LiTFSI) coated on a polypropylene membrane. PEO is a polymer matrix that is commonly used to prepare gel polymer electrolytes [34] and is coated on the polypropylene membrane to inhibit the polysulfide diffusion and improve cell stability. The LiTFSI salt contributes fast lithium-ion transfer capability to the PEO coating, thereby improving its electrochemical efficiency. To demonstrate that this functional separator can block polysulfides, the separator is directly assembled with a polysulfide cathode and then tested. The performance of the developed cathodes is compared against that of a similar PEO-coated polypropylene membrane that lacks the fast lithium-ion transfer capability (i.e., the reference cell) and a regular separator that lacks the polysulfide-blocking capability (i.e., the control cell).

## 2. Materials and Methods

### 2.1. Fabrication of PEO/LiTFSI-Coated Polypropylene Membrane

Poly(ethylene oxide) (PEO, M_w_ = 600,000, Sigma Aldrich, St. Louis, MO, USA) and lithium bis(trifluoromethanesulfonyl)imide (LiTFSI, 99.95%, Sigma Aldrich) at a molar ratio of O/Li at 20:1 were mixed in acetonitrile (99%, J.T. Baker) and stirred for 24 h to form a transparent gel polymer electrolyte. The well-mixed gel polymer electrolyte was cast as the functional coating material onto a polypropylene membrane (Celgard, Summit, NJ, USA), and the coated membrane was then dried in a vacuum oven at 80 °C for 24 h. The as-prepared PEO/LiTFSI-coated polypropylene membrane was cut into circular disks, 19 mm in diameter. As a reference membrane for investigating lithium-ion diffusion issues, a PEO-coated polypropylene membrane was fabricated using the same procedure, but without LiTFSI. Finally, a polypropylene membrane was prepared for assembling control cells.

### 2.2. Physicochemical Characterization

The phase and crystal structures of PEO, LiTFSI, the polypropylene membrane, the PEO-coated polypropylene membrane, and the PEO/LiTFSI-coated polypropylene membrane were characterized by X-ray diffraction (XRD, D8 Discover, Bruker, Billerica, MA, USA). The morphology and microstructure of the polypropylene membranes with and without coating layers were observed by field-emission scanning electron microscopy (FE-SEM, JSM-7001, JEOL, Tokyo, Japan).

### 2.3. Electrochemical Characterization and Cell Performance

The electrochemical characteristics and cell performance of the PEO/LiTFSI-coated polypropylene membrane were analyzed by fabricating electrochemical lithium–sulfur cells. A 1.0 M polysulfide catholyte was obtained by mixing sulfur (99.5%, Alfa Aesar, Haverhill, MA, USA) and lithium sulfide (99+%, Alfa Aesar) into a blank electrolyte solution composed of 1.85 M LiTFSI (99.95%, Sigma Aldrich) and 0.2 M LiNO_3_ (99.98%, Alfa Aesar) dissolved in mixed 1,2-dimethoxyethane (99+%, Alfa Aesar) and 1,3-dioxacyclopentane (99+%, Alfa Aesar) solution. The polysulfide catholyte was added to a battery current collector to fabricate a polysulfide cathode at a fixed sulfur loading of 2 mg cm^−2^ and sulfur content of 51 wt % (considering the weight of the whole cathode). Electrochemical lithium–sulfur cells were assembled in an argon-filled glove box by combining the polysulfide cathode, the PEO/LiTFSI-coated polypropylene membrane, and a lithium counter electrode. For the performance comparison, reference and control cells were assembled using a PEO-coated polypropylene membrane and an uncoated separator, respectively. High-loading analysis was conducted on high-loading polysulfide cathodes with sulfur loadings (contents) of 4 mg cm^−2^ (67 wt %) and 6 mg cm^−2^ (76 wt %), respectively. The assembled lithium–sulfur cells were allowed to rest at 25 °C for 30 min before electrochemical cycling and analysis. Electrochemical impedance spectroscopy data were obtained from 1 MHz to 100 mHz using an impedance analyzer (SP-150, Biologic, Seyssinet-Pariset, France) at an AC voltage amplitude of 5 mV and open-circuit voltage. Cyclic voltammograms (CV) were recorded using a potentiostat (VMP-300, Biologic) in the voltage window of 1.8 to 2.8 V at scan rates of 0.02, 0.03, 0.04, and 0.05 mV s^−1^. Charge/discharge voltage profiles, cycling performance data, polarization analysis, and high-loading performance data were collected in the same range voltage window using a programmable battery cycler (BCS-800, Biologic) at a regular cycling rate of C/10 (1C = 1675 mA g^−1^). The cycling capacity and high-loading performance of cells were evaluated on the basis of the weight of sulfur.

## 3. Results and Discussion

### 3.1. Material Properties of the Functional Membranes

**Figure 1:**
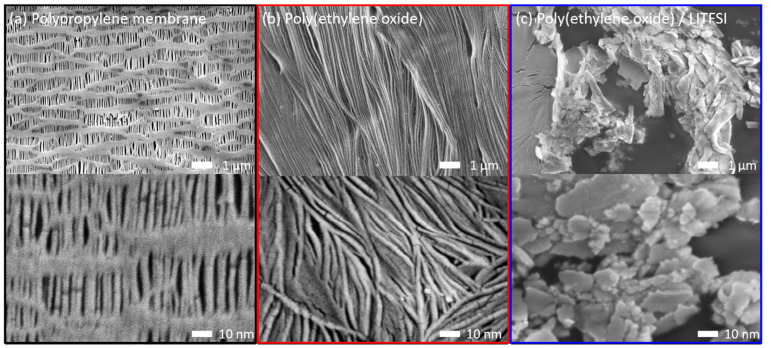
Morphology and microstructure of the (**a**) polypropylene membrane (black box), (**b**) PEO-coated polypropylene membrane (red box), and (**c**) PEO/LiTFSI-coated polypropylene membrane (blue box). PEO = poly(ethylene oxide) and LiTFSI = lithium bis(trifluoromethanesulfonyl)imide.

Figure 1 depicts the surface morphology of the polypropylene membrane, PEO-coated polypropylene membrane, and PEO/LiTFSI-coated polypropylene membrane. The uncoated polypropylene membrane with an average pore size of 0.06 µm (Figure 1a) has a thickness of 25 µm and a weight of 1.43 mg cm^−2^. The PEO-coated polypropylene membrane has a uniform PEO coating (Figure 1b), showing a thickness of 29 µm and a weight of 2.20 mg cm^−2^. The PEO/LiTFSI-coated polypropylene membrane exhibits a deposition of LiTFSI salts on the PEO matrix (Figure 1c), having a thickness of 31 µm and a weight of 3.55 mg cm^−2^. The PEO and PEO/LiTFSI coatings are configured toward the polysulfide cathode such that they would be able to block the fast and irreversible polysulfide diffusion during lithium–sulfur cell cycling [10,35]. The PEO/LiTFSI coating differs from the PEO coating as the former forms a gel polymer electrolyte that attaches to the polypropylene membrane to further give the smooth lithium-ion transfer between the two electrodes [10,36]. After being placed in an electrolyte solution, the PEO and PEO/LiTFSI coatings still bond to the polypropylene membrane, suggesting the mechanical strength of the coated separator.

Figure S1 depicts the XRD spectra of the coating materials (i.e., PEO, LiTFSI, and PEO/LiTFSI); the pure phases of PEO and LiTFSI can be seen in Figure S1a,b. In Figure S1c, the low concentration of LiTFSI is evident by the absence of XRD peaks for this species, which might also be masked by the strong XRD peaks of PEO. Figure S2 presents the XRD spectra of the tested membranes. The uncoated separator shows polypropylene XRD peaks (Figure S2a). The coated membranes display overlapping polypropylene and PEO XRD patterns, whereas the XRD peaks of LiTFSI are relatively weak and might be somewhat masked by the strong PEO peaks (Figure S2b,c).

### 3.2. Electrochemical Characteristics and Cell Performance of Cells with Functional Membranes

**Figure 2:**
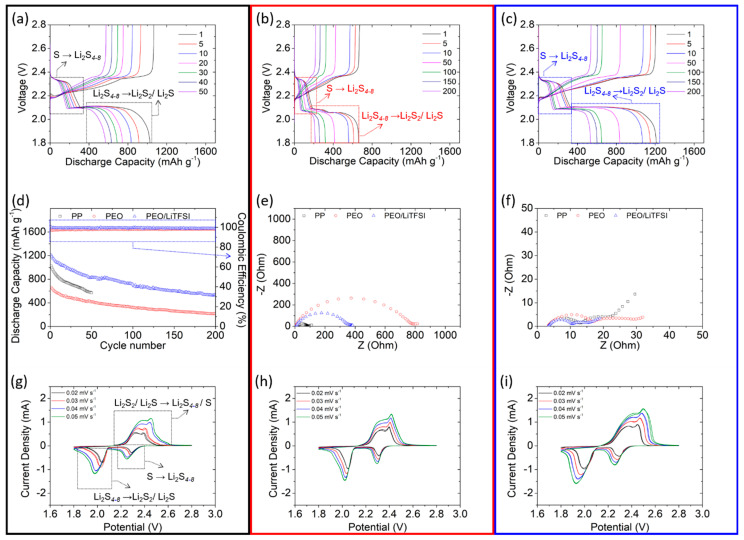
Electrochemical characteristics and cell performance of lithium–sulfur cells with different membranes: discharge/charge curves of the (**a**) polypropylene membrane, (**b**) PEO-coated polypropylene membrane, and (**c**) PEO/LiTFSI-coated polypropylene membrane. (**d**) Cycling performance, (**e**) fresh-cell impedance, (**f**) cycled-cell impedance, and rate-dependent CV analysis of the cells with the (**g**) polypropylene membrane, (**h**) PEO-coated polypropylene membrane, and (**i**) PEO/LiTFSI-coated polypropylene membrane. PEO = poly(ethylene oxide) and LiTFSI = lithium bis(trifluoromethanesulfonyl)imide.

Figure 2 illustrates the electrochemical characteristics and the lithium–sulfur cell performance of the tested membranes. We use the PEO/LiTFSI-coated polypropylene membrane in the experimental cell (marked as a blue box) to demonstrate the membrane’s enhanced electrochemical efficiency and stability, the PEO-coated polypropylene membrane in the reference cell (marked as a red box) to demonstrate the issue of lithium-ion transfer in the coating layer, and a regular, unmodified membrane in the control cell (marked as a black box). Moreover, to study the effectiveness of the functional membranes in solving the polysulfide-related problems, the electrochemical cells are fabricated and configured to face a polysulfide cathode.

Figure 2a presents the charge/discharge voltage profiles of the control cell, in which the typical two-step redox reaction of lithium–sulfur cells are evident. The two distinguishable discharge plateaus from 2.3 to 1.8 V indicate the two complete reduction reactions: from solid-state sulfur to liquid-state polysulfides (Li_2_S*_x_*, 4 < *x* ≤ 8) at 2.3–2.1 V and, subsequently, from liquid-state polysulfides to solid-state sulfides (Li_2_S*_x_*, *x* = 1, 2) at 2.1–1.8 V [3,37]. The two continuous charge plateaus from 2.2 to 2.8 V indicate the reversible oxidation reaction from sulfide to polysulfides and sulfur at 2.2–2.3 V and 2.3–2.8 V, respectively [3,38]. The control cell undergoes fast capacity fade and has poor electrochemical efficiency, resulting in a short cycle life of 50 cycles and low capacity retention (55%), highlighting the severe consequences of uncontrolled polysulfide relocation and the resulting irreversible redeposition of the insulating solid-state active material in the cell [39]. Figure 2b presents the charge/discharge voltage profiles of the reference cell, which has a PEO-coated membrane that hinders the fast diffusion of dissolved polysulfides: as can be seen, the PEO coating extends the cycle life to 200 cycles. Although the PEO coating is applied as a polymer matrix in the gel polymer electrolyte, its low lithium-ion transfer capability results in low Coulombic efficiency and low electrochemical utilization in the cell [3,10,40]. Figure 2c depicts the charge/discharge voltage profiles of the PEO/LiTFSI-coated polypropylene membrane; it features overlapping discharge and charge curves over 200 continuous cycles, suggesting improved capacity retention. This high electrochemical stability indicates that the PEO/LiTFSI coating stabilizes the dissolved polysulfides within the cathode region of the cell [10,41]. The enhanced electrochemical efficiency and reversibility suggests that the coated PEO/LiTFSI film functions as a gel polymer electrolyte that ensures smooth lithium-ion transfer and hence a long cycle life with low polarization (Figure S3). Thus, the lithium–sulfur cell fabricated with the PEO/LiTFSI-coated polypropylene membrane exhibits a high charge-storage capacity of 1212 mA∙h g^−1^ and long cyclability, with a high reversible capacity of 534 mA∙h g^−1^ and stable Coulombic efficiency of >99% over 200 cycles (Figure 2d). The reference cell also shows a long cycle life due to the stabilization of polysulfides within the cathode region. However, the low conductivity of the PEO polymer matrix slows the reaction kinetics. This results in low electrochemical utilization and a low discharge capacity of 662 mA∙h g^−1^, which decreases to 214 mA∙h g^−1^ after cycling. The control cells, in contrast, have a short cycle life of 50 cycles due to the fast polysulfide diffusion that causes the irreversible capacity loss.

Figure 2e,f presents a comparison of the impedance of the cells with the tested membranes before and after cycling. In uncycled cells, the coated membranes produce higher charge-transfer impedance and the PEO coating shows the highest impedance due to the insulating nature of PEO at room temperature (Figure 2e). After cycling, the cells exhibit a relatively low charge-transfer impedance and cell resistance compared to those of uncycled cells (Figure 2f). This decrease in impedance is attributable to the relocation of the physically stable active material, which occupies a more electrochemically favorable position in the cathode substrate after the first cycle [42]. Figure 2g–i presents the CV analysis of the tested cells, illustrating their electrochemical reversibility and polarization, in addition to their lithium-ion diffusion coefficients (calculated using the relationship of the peak current and the CV scanning rate). The scanning rate varies from 0.02 to 0.05 mV s^−1^ with repeated tests (three scans at each rate) (Figures S4–S6). The repeated scans at various rates yield overlapping curves in the cell with the coated membranes, demonstrating the excellent reversibility of the cathode redox reactions [43]. Next, we compare the rate-dependent CV curves at 0.02–0.05 mV s^−1^. As the scanning rate increases, the voltage delay caused by the fast reaction rates leads to electrochemical polarization. The additional charge-transfer impedance contributed by the PEO coating, and the PEO/LiTFSI coating only leads to slightly high polarization. The high lithium-ion diffusion coefficient of the PEO/LiTFSI coating (9.6 × 10^−9^–3.0 × 10^−8^ cm^2^ s^−1^; Figure S6f) demonstrates that the coating improves lithium-ion transfer. In contrast, the reference and control cells have low diffusion coefficients of 7.6 × 10^−9^–2.2 × 10^−8^ cm^2^ s^−1^ and 5.6 × 10^−9^–2.8 × 10^−8^ cm^2^ s^−1^ due to the obstruction of the PEO coating and the lack of ionic conductive channels, respectively (Figures S4f and S5f). The aforementioned results indicate that PEO/LiTFSI-coated polypropylene membranes block the fast migration of polysulfides from the cathode, while allowing smooth lithium-ion transfer. These advantages will enable the use of polysulfide catholytes in electrochemical lithium–sulfur cells and spur the development of high-loading sulfur cathodes with high energy density, fast reaction kinetics, and reversibility.

### 3.3. Electrochemical and Cell Performance of Functional Membranes for High-Loading Cathodes

**Figure 3:**
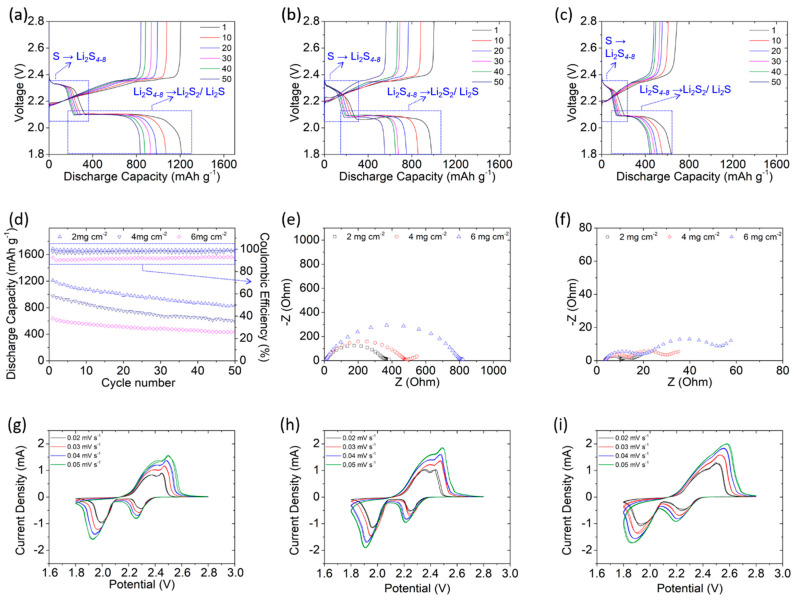
Electrochemical and cell performance of high-loading sulfur cathodes with a PEO/LiTFSI-coated polypropylene membrane: Discharge/charge curves of the cathode with sulfur loadings of (**a**) 2 mg cm^−2^, (**b**) 4 mg cm^−2^, and (**c**) 6 mg cm^−2^. (**d**) Cycling performance, (**e**) fresh-cell impedance, (**f**) cycled-cell impedance, and rate-dependent CV analysis of cells with sulfur loadings of (**g**) 2 mg cm^−2^, (**h**) 4 mg cm^−2^, and (**i**) 6 mg cm^−2^. PEO = poly(ethylene oxide) and LiTFSI = lithium bis(trifluoromethanesulfonyl)imide.

Figure 3a–c presents the voltage profiles of the polysulfide cathodes with PEO/LiTFSI-coated polypropylene membranes at sulfur loadings of 2 mg cm^−2^, 4 mg cm^−2^, and 6 mg cm^−2^ (sulfur contents = 51, 67, and 76 wt%). Despite the increase in the amount of active material in the cathode, the discharge and charge curves maintain distinguishable upper and lower discharge plateaus, in addition to two continuous charge plateaus. The polarization is also maintained low (Figure S7). These results indicate that the PEO/LiTFSI-coated polypropylene membrane affords high-loading sulfur cathodes with good electrochemical reaction capability and high polysulfide retention [3,8,9,44]. Figure 3d illustrates the cycling performance of the high-loading polysulfide cathodes, which attain high discharge capacities of 1212 mA∙h g^−1^, 981 mA∙h g^−1^, and 637 mA∙h g^−1^ at sulfur loadings (contents) of 2 mg cm^−2^ (51 wt%), 4 mg cm^−2^ (67 wt %), and 6 mg cm^−2^ (76 wt %), respectively. After cycling, these high-loading cathodes maintain a high-capacity retention of 60%–70% and attain a high areal capacity of 2.4–4.0 mA∙h cm^−2^. Thus, the PEO/LiTFSI composite is a functional coating that facilitates good polysulfide retention and smooth lithium-ion transfer. Moreover, a polysulfide cathode with the developed PEO/LiTFSI-coated polypropylene membrane can host a large amount of sulfur without sacrificing its high electrochemical utilization and efficiency.

To verify the foregoing results, the electrochemical reaction kinetics (Figure 3e,f) and reversibility/stability (Figure 3g–i) of high-loading sulfur cathodes are evaluated from their impedance spectra and rate-dependent CV curves, respectively. In Figure 3e,f, the low and decreasing cell impedance featuring low charge-transfer resistance and ion-diffusion impedance of the cell after cycling indicate good reaction capability and fast ion transfer [44,45]. Figure 3g–i presents the rate-dependent CV curves of the cells with sulfur loadings (content) of 2 mg cm^−2^ (51 wt%), 4 mg cm^−2^ (67 wt %), and 6 (76 wt %) mg cm^−2^, respectively. Despite increasing sulfur loadings, the cells display similar redox reaction peaks with no obvious voltage delays and rapid current drops, illustrating their good electrochemical reversibility. However, the lithium-ion diffusion coefficients decrease slightly from 1.5 × 10^−8^–5.0 × 10^−8^ cm^2^ s^−1^ to 1.2 × 10^−8^–3.8 × 10^−8^ cm^2^ s^−1^ with increasing sulfur loading (Figures S8–S10) because an increase in the amount of sulfur and polysulfide increases the thickness of the electrode and the viscosity of the electrolyte, possibly delaying lithium-ion transfer. Nevertheless, this increase in thickness does not affect the cell’s electrochemical efficiency, and the overlapping CV curves and similar lithium-ion diffusion coefficients verify the cell’s high electrochemical stability.

## 4. Conclusions

We develop a functional separator fabricated using a thin-film gel polymer electrolyte and a robust polypropylene membrane. Given its excellent polysulfide-blocking capability, the PEO/LiTFSI-coated polypropylene membrane is configured to face the polysulfide cathode, while the added LiTFSI serves as an ion transfer network. Our tests reveal that this cell has low cell resistance and overlapping discharge/charge curves before and after cycling, indicating its improved reaction kinetics and electrochemical utilization. Moreover, the overlapping CV curves and the high lithium-ion diffusion coefficients indicate that the cell exhibits smooth ion transfer and enhanced electrochemical reversibility and stability. As a result, a high-loading polysulfide cathode fabricated using the developed membrane at a high sulfur loading of 6 mg cm^−2^ and high sulfur content of 76 wt % has a high areal specific capacity of 4 mA∙h cm^−2^. In contrast, control and reference cells fabricated using a PEO-coated polypropylene membrane and a regular separator membrane, respectively, exhibit low ion transfer and fast polysulfide relocation, resulting in low charge-storage capacities and short cycle lives. Our findings highlight the importance of the proposed design in developing advanced functional separators for lithium–sulfur batteries.

## Data Availability

The data presented in this study are available in this study and the corresponding supplementary material.

## Supplementary Materials

The following are available online at https://www.mdpi.com/2073-4360/13/4/535/s1, Figures S1 and S2: X-ray diffraction (XRD) analysis, Figures S3–S6: Polarization and CV of lithium–sulfur cells, Figures S7–S10: Polarization and CV of the lithium–sulfur cells using the PEO/LiTFSI-coated polypropylene membrane.
