# Supplementary material for: A Poly(ethylene oxide)/Lithium bis(trifluoromethanesulfonyl)imide-Coated Polypropylene Membrane for a High-Loading Lithium–Sulfur Battery
Source: Polymers (Basel). 2021 Feb 11;13(4):535. doi: 10.3390/polym13040535 (PMC7918845; doi:10.3390/polym13040535)
Supplement: Supplementary file 1 [file polymers-13-00535-s001.pdf]

polymers

MDPI

Supporting Information

A Poly(ethylene oxide)/Lithium bis(trifluoromethanesulfonyl)imide-Coated Polypropylene Membrane for a High-Loading Lithium–Sulfur Battery

Li-Ling Chiu [1] and Sheng-Heng Chung [1,2,*]

[1] Department of Materials Science and Engineering, National Cheng Kung University; N56094570@gs.ncku.edu.tw (L.-L.C.); SHChung@gs.ncku.edu.tw (S.-H.C.)
[2] Hierarchical Green-Energy Materials Research Center, National Cheng Kung University; SHChung@gs.ncku.edu.tw (S.-H.C.)
* Correspondence: SHChung@gs.ncku.edu.tw (S.-H.C.)

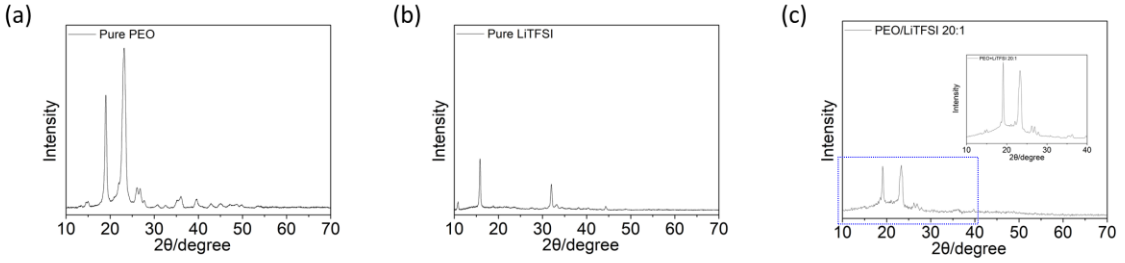

Figure S1. X-ray diffraction (XRD) analysis of: (a) PEO, (b) LiTFSI, and (c) PEO/LiTFSI. PEO = poly(ethylene oxide) and LiTFSI = lithium bis(trifluoromethanesulfonyl)imide.

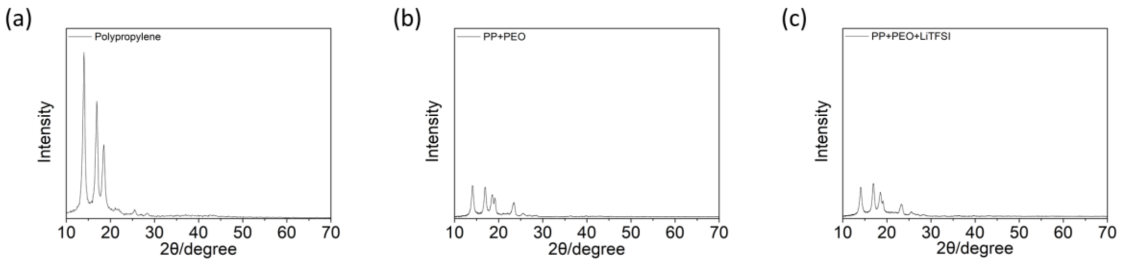

Figure S2. XRD analysis of: (a) polypropylene membrane, (b) PEO-coated polypropylene membrane, and (c) PEO/LiTFSI-coated polypropylene membrane.

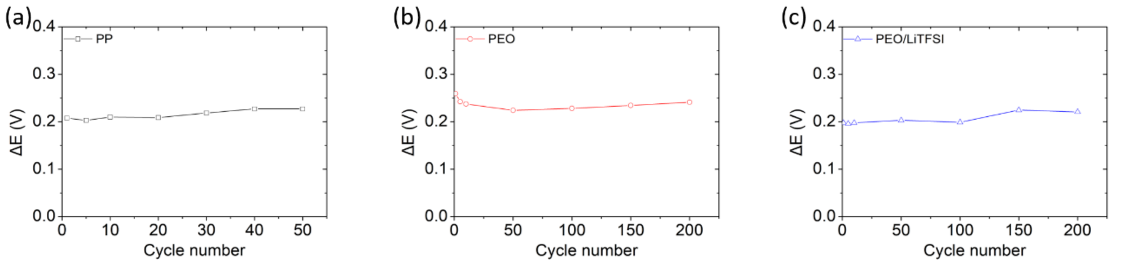

Figure S3. Polarization of lithium-sulfur cells with (a) polypropylene membrane, (b) PEO-coated polypropylene membrane, and (c) PEO/LiTFSI-coated polypropylene membrane.

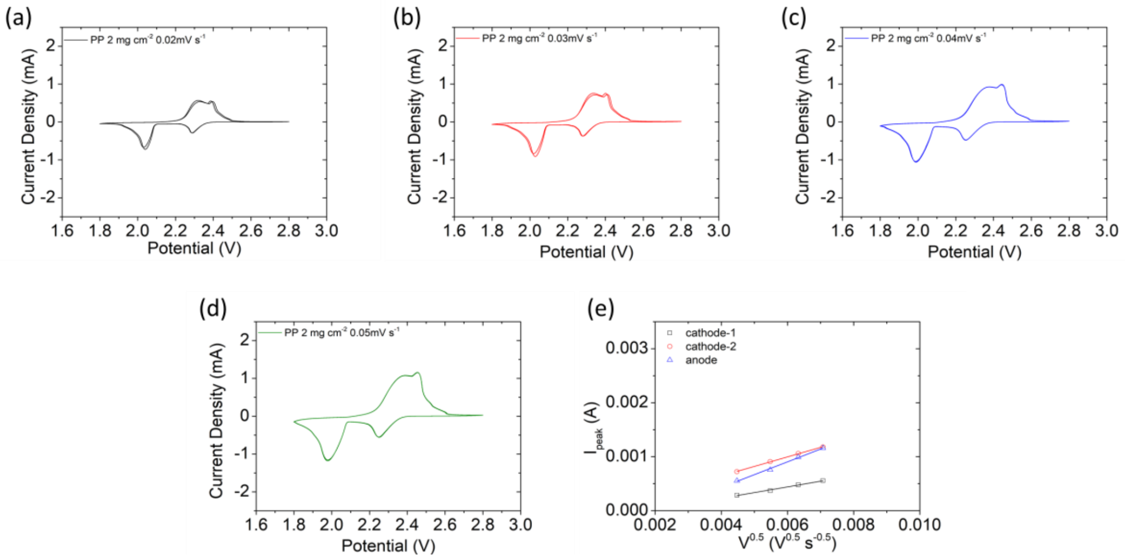

Figure S4. CV scanning curves of lithium-sulfur cells with a polypropylene membrane at (a) 0.02. (b) 0.03, (c) 0.04, and (d) 0.05 mV s^{-1}. (e) Lithium-ion diffusion coefficient analysis.

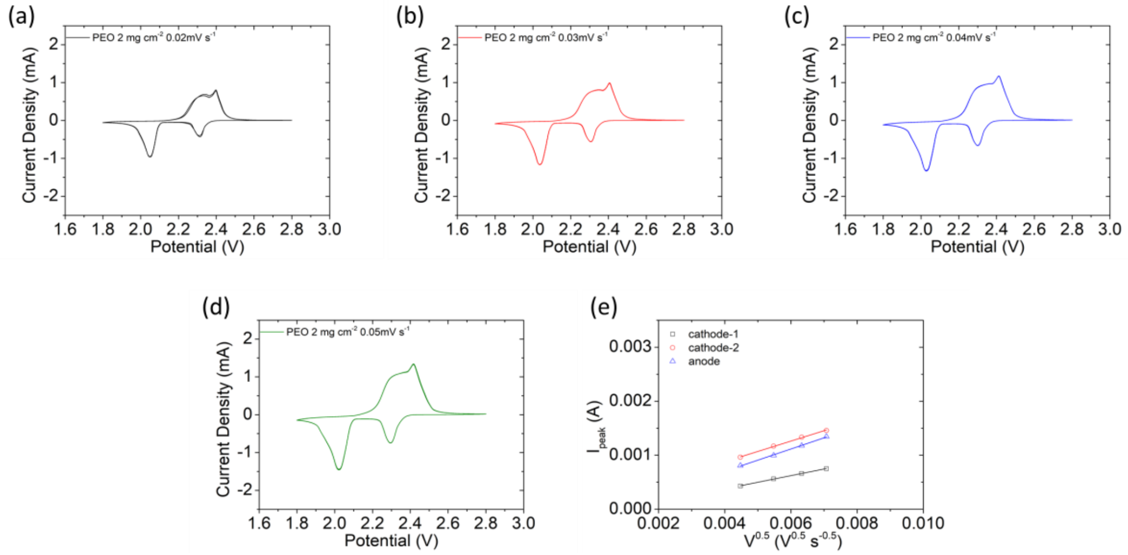

Figure S5. CV scanning curves of lithium-sulfur cells with a PEO-coated polypropylene membrane at (a) 0.02. (b) 0.03, (c) 0.04, and (d) 0.05 mV s^{-1}. (e) Lithium-ion diffusion coefficient analysis.

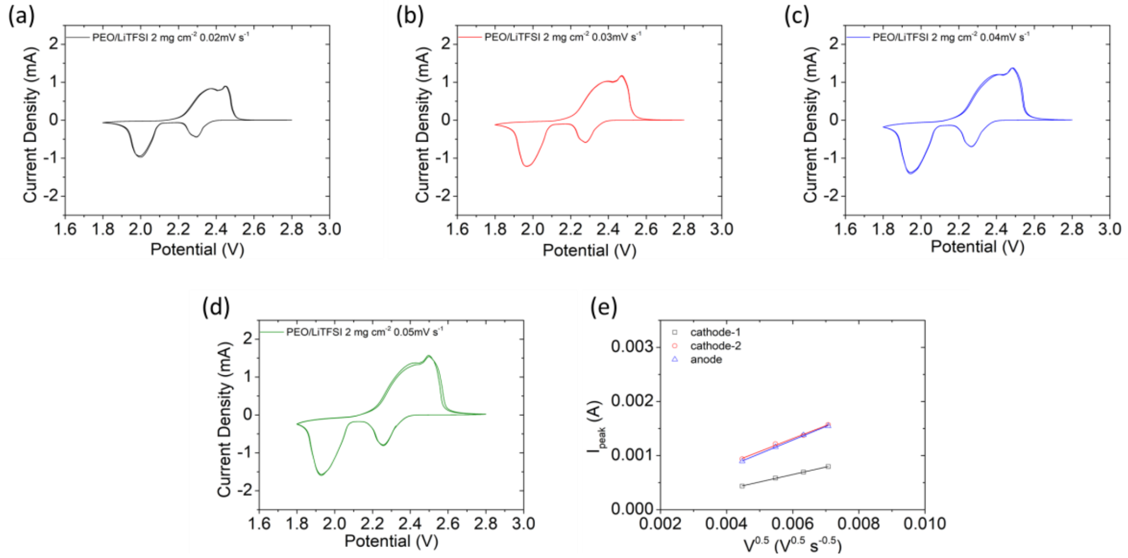

Figure S6. CV scanning curves of lithium-sulfur cells with a PEO/LiTFSI-coated polypropylene membrane at (a) 0.02. (b) 0.03, (c) 0.04, and (d) 0.05 mV s^{-1}. (e) Lithium-ion diffusion coefficient analysis.

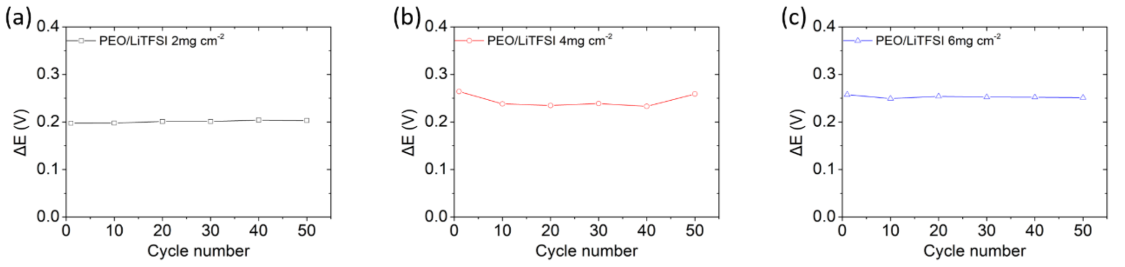

Figure S7. Polarization of lithium-sulfur cells using a PEO/LiTFSI-coated polypropylene membrane with different sulfur loadings: (a) 2 mg cm^{-2}, (b) 4 mg cm^{-2}, and (c) 6 mg cm^{-2}.

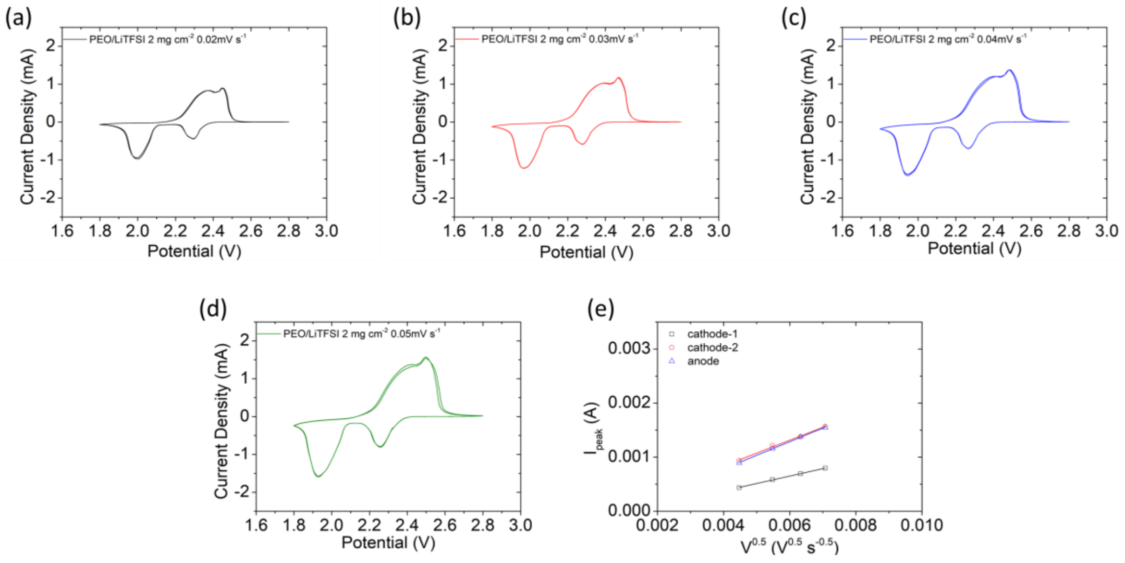

Figure S8. CV scanning curves of lithium-sulfur cells using a PEO/LiTFSI-coated polypropylene membrane with a sulfur loading of 2 mg cm^{-2} and a sulfur content of 51 wt% at (a) 0.02. (b) 0.03, (c) 0.04, and (d) 0.05 mV s^{-1}. (e) Lithium-ion diffusion coefficient analysis.

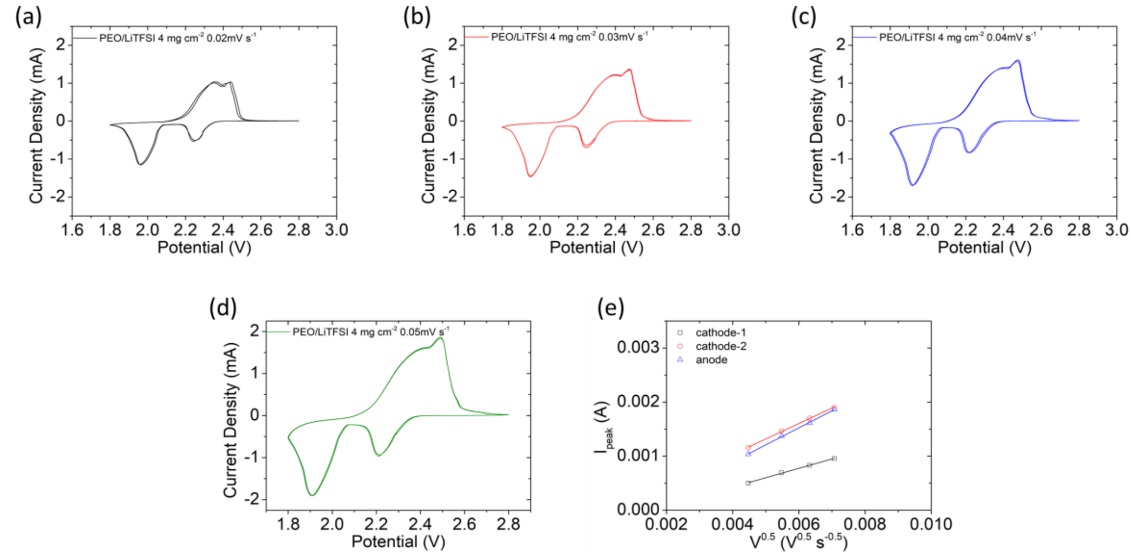

Figure S9. CV scanning curves of lithium-sulfur cells with a PEO/LiTFSI-coated polypropylene membrane with a sulfur loading of 4 mg cm^{-2} and a sulfur content of 67 wt% at (a) 0.02. (b) 0.03, (c) 0.04, and (d) 0.05 mV s^{-1}. (e) Lithium-ion diffusion coefficient analysis.

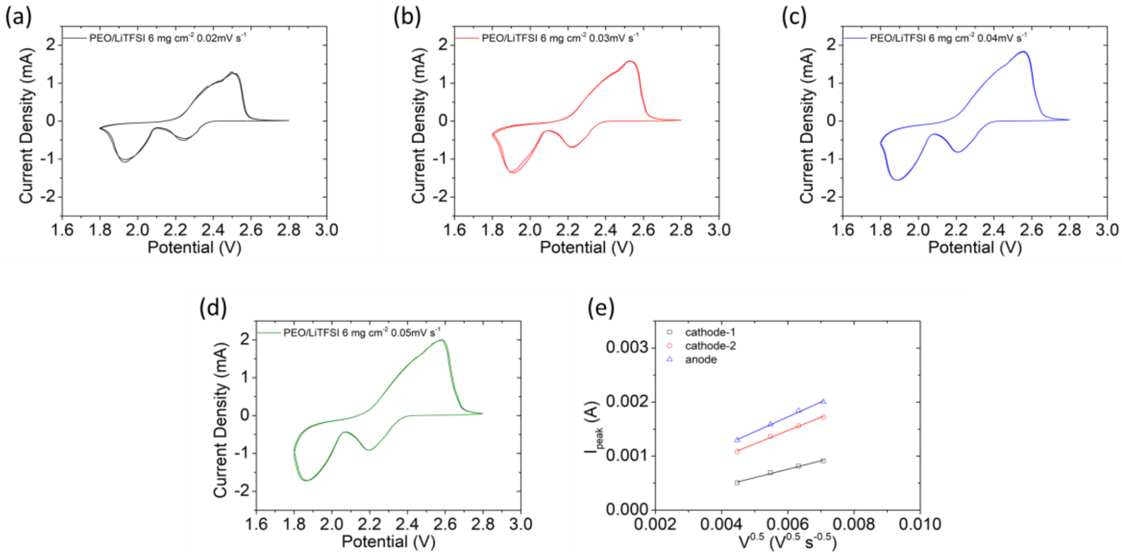

Figure S10. CV scanning curves of lithium-sulfur cells using a PEO/LiTFSI-coated polypropylene membrane with a sulfur loading of 6 mg cm^{-2} and a sulfur content of 76 wt% at (a) 0.02. (b) 0.03, (c) 0.04, and (d) 0.05 mV s^{-1}. (e) Lithium-ion diffusion coefficient analysis.
